# Uncovering the burden of Influenza in children in Portugal, 2008–2018

**DOI:** 10.1186/s12879-023-08685-z

**Published:** 2024-01-18

**Authors:** Alberto Caldas Afonso, Catarina Gouveia, Gustavo Januário, Mafalda Carmo, Hugo Lopes, Hélène Bricout, Catarina Gomes, Filipe Froes

**Affiliations:** 1 Unidade de Nefrologia Pediátrica, Centro Materno-Infantil do Norte, Centro Hospitalar Universitário do Porto, Porto, Portugal; 2 Centro Hospitalar Universitário Santo António, Instituto de Ciências Biomédicas Abel Salazar, Porto, Portugal; 3 EPIUnit - Instituto de Saúde Pública, Porto, Portugal; 4 Laboratório para a Investigação Integrativa e Translacional em Saúde Populacional, Porto, Portugal; 5 Hospital D. Estefânia, Centro Hospitalar Lisboa Central, Lisboa, Portugal; 6 Faculdade de Ciências Médicas, Nova Medical School, Lisbon, Portugal; 7 Hospital Pediátrico, Centro Hospitalar e Universitário de Coimbra, Coimbra, Portugal; 8 Faculdade de Medicina, Universidade de Coimbra, Coimbra, Portugal; 9 IQVIA, Barcelona, Spain; 10 IQVIA, Lisbon, Portugal; 11 NOVA National School of Public Health, Public Health Research Centre, Universidade NOVA de Lisboa, Lisbon, Portugal; 12 Comprehensive Health Research Center - Universidade NOVA de Lisboa, Lisbon, Portugal; 13 Sanofi, Lyon, France; 14 Sanofi, Lisbon, Portugal; 15 Hospital Pulido Valente, Centro Hospitalar Universitário Lisboa Norte, Lisbon, Portugal

**Keywords:** Influenza, Burden, Excess, Mortality, Hospitalization, Children, Healthy, Portugal

## Abstract

**Background:**

Despite their higher risk of developing severe disease, little is known about the burden of influenza in Portugal in children aged < 5 years old. This study aims to cover this gap by estimating the clinical and economic burden of severe influenza in children, in Portugal, during ten consecutive influenza seasons (2008/09-2017/18).

**Methods:**

We reviewed hospitalizations in children aged < 5 years old using anonymized administrative data covering all public hospitals discharges in mainland Portugal. The burden of hospitalization and in-hospital mortality directly coded as due to influenza was supplemented by the indirect burden calculated from excess hospitalization and mortality (influenza-associated), estimated for four groups of diagnoses (pneumonia or influenza, respiratory, respiratory or cardiovascular, and all-cause), through cyclic regression models integrating the incidence of influenza. Means were reported excluding the H1N1pdm09 pandemic (2009/10).

**Results:**

The mean annual number of hospitalizations coded as due to influenza was 189 (41.3 cases per 100,000 children aged < 5 years old). Hospitalization rates decreased with increasing age. Nine-in-ten children were previously healthy, but the presence of comorbidities increased with age. Children stayed, on average, 6.1 days at the hospital. Invasive mechanical ventilation was used in 2.4% of hospitalizations and non-invasive in 3.1%. Influenza-associated excess hospitalizations between 2008 and 2018 were estimated at 1,850 in pneumonia or influenza, 1,760 in respiratory, 1,787 in respiratory or cardiovascular, and 1,879 in all-cause models. A total of 95 influenza-associated excess deaths were estimated in all-cause, 14 in respiratory or cardiovascular, and 9 in respiratory models. Over ten years, influenza hospitalizations were estimated to have cost the National Health Service at least €2.9 million, of which 66.5% from healthy children.

**Conclusions:**

Influenza viruses led to a high number of hospitalizations in children. Most were previously healthy. Results should lead to a reflection on the adequate preventive measures to protect this age group.

**Supplementary Information:**

The online version contains supplementary material available at 10.1186/s12879-023-08685-z.

## Background

Every year, influenza viruses are estimated to infect between 5 and 10% of adults and 20–30% of children across the globe, causing substantial morbidity and mortality [1]. Influenza’s clinical manifestations can range from asymptomatic to severe illness, often [2]triggering complications in children, such as primary viral or secondary bacterial pneumonia, seizures, sinusitis, acute otitis media, exacerbations of existing respiratory conditions, amongst others [2–4].

Although [1]annual influenza vaccination in children aged 6–59 months is recommended by the World Health Organization, some countries – such as Portugal – recommend it only for high-risk children [3, 5–8]. There are few studies in Europe focused on understanding the burden of influenza in children and characterizing the severe cases that lead to hospitalization and even death [9, 12], despite evidence suggesting that children aged < 5 years old may have the highest hospitalizations rates amongst all age groups [10].

In our study, we aimed to estimate the epidemiological and economic burden of severe influenza in children aged < 5 years old in Portugal, during ten consecutive influenza seasons (2008/09-2017/18). Secondary objectives included the description of the clinical and demographic characteristics of hospitalized children, as well as outcomes and severity indicators of the episodes. The burden of hospitalization and in-hospital mortality directly due to influenza (i.e., coded as due to influenza) as calculated from the National Health Service (NHS) discharge data was supplemented by the burden calculated from excess hospitalization and mortality indirectly due to influenza (influenza associated) and related costs.

## Methods

The Burden of Acute Respiratory Infections (BARI) study is a multidimensional real-world evidence study assessing the clinical and economic burden of acute respiratory infections (influenza and respiratory syncytial virus) in Spain and Portugal [10–15]. Here we report results for the burden of severe influenza in children aged < 5 years old in Portugal, measured through hospitalizations and deaths. Results for other age groups are reported by Froes et al. (2022) [10].

The following approach was used: (i) a direct method of estimating seasonal influenza incidence, based on the number of NHS hospitalizations with influenza-specific International Classification of Diseases (ICD) codes; (ii) an indirect method of estimating excess hospitalizations and deaths using broader groups of ICD codes in time-series ecological models [10].

### Data sources

#### Hospital discharge data

Anonymized administrative data on hospitalizations (January 2008–December 2018) were provided by the *Administração Central do Sistema de Saúde* (Health System Central Administration), which collects administrative and clinical data for all hospitalization episodes in Portuguese public hospitals, including information on diagnoses and procedures performed during hospital stay, which are coded using the ICD-9-CM and ICD-10-CM/PCS. These data were also used to describe the in-hospital case fatality risk based on discharge status, corresponding to the hospitalization episodes coded as due to influenza that resulted in in-hospital death during the respective episode.

### Death certificate data

The analysis of influenza-associated excess mortality used data on daily deaths listed in the death certificate for children aged < 5 years old per cause obtained from the *Instituto Nacional de Estatística* (INE, National Statistics Institute) in July 2020. The data were aggregated into weekly counts [16]. The coding for primary death from the death certificate was used.

### Influenza activity data

The primary predictor of influenza excess hospitalizations and deaths was the overall weekly incidence rate of influenza-like-illness (ILI), which was obtained from the *Instituto Nacional de Saúde Doutor Ricardo Jorge* (National Institute of Health Doutor Ricardo Jorge) in July 2020 [17].

### Demographic data

Data on age-specific annual resident population estimates were downloaded from INE’s website [16] to compute rates of cases per 100,000 people.

### Statistical analysis

An ecological approach was used to estimate the number of influenza-associated excess hospitalizations, deaths, and hospitalization costs by using different Poisson cyclic regression models (time series), where cause-specific hospitalization and mortality data were explained by the ILI incidence, as well as time trends and seasonal terms, using a log link (moving average, broken down by season, with a one-week lag for hospitalizations and three-week lag for deaths) [10, 11, 18].

The number of influenza-associated hospitalizations or deaths was defined for each epidemic season by the sum of the weekly excesses. The seasons were defined from September to June of each year, as standard for the Northern Hemisphere. Cases per 100,000 people were computed by dividing the estimated influenza cases by the Portuguese population aged < 5 years old. Means were reported only for nine seasons, excluding the H1N1pdm09 pandemic (2009/10). Three-year means, including the most recent seasons (2015/16 to 2017/18), were also included.

The correlation between the values predicted by the model and those observed indicated the performance of the model, using Pearson’s correlation. The mean absolute percentage error, defined as the average of the percentage difference between predicted and observed values for all weeks, was also computed. All analyses were performed using SAS 9.4.

### Case definition

#### Hospitalizations coded as due to Influenza

Data were extracted from administrative databases from 2008 to 2018 that contained all NHS hospitalization records in mainland Portugal for children aged < 5 years old. Influenza episodes were defined as those coded with ICD-9 487 or 488; or ICD-10 J09, J10, or J11 in any primary or secondary diagnosis field [10, 19]. Diagnostics information during the influenza episode was also used to identify children who had at least one medical condition regarded as a risk factor for severe influenza (respiratory or lung disease, cardiovascular disease, immunocompromised, and other conditions – Down syndrome, diabetes mellitus, chronic liver/kidney disease, neuromuscular impairment), in any primary or secondary diagnosis field, as detailed in Table S1.

Mechanical ventilation was used as surrogate indicator of Intensive Care Unit (ICU) admissions, as these are not separately identified in the database [14, 20].

### Excess hospitalization and mortality

Influenza-associated excess hospitalization was computed for four groups of diagnoses, according to the primary diagnosis, namely pneumonia or influenza (P&I, ICD-9: 480–488, 517.1; or ICD-10: J09–J18), respiratory (R, ICD-9: 460–519; or ICD-10: J00–J99), respiratory or cardiovascular (R&C, ICD-9: 390–459, 460–519; or ICD-10: I00–I99, J00–J99), and all-cause (any ICD-9/10 diagnosis). Influenza-associated excess mortality was computed for the same groups of diagnoses according to the cause of death listed on the death certificate.

### Cost estimation

Only direct costs were estimated using a diagnosis-related group-based budget allocation model. Hospitalization costs were computed by multiplying each cost weight with the Portuguese fixed cost multiplier and funding price applicable for the 2018 year [10]. The cost of excess hospitalization was computed by multiplying the number of estimated excess hospitalizations by the mean cost per hospitalization by cause.

### Ethical considerations

The study was conducted following the ethical principles of the Declaration of Helsinki and as per local regulations, including privacy laws. Data were provided anonymized and may be used for research purposes without the approval of an ethics committee or informed consent. In addition, the protocol of the BARI study was validated by a panel of clinical experts, classified by the Agency of Medicines and Medical Devices as an observational study and approved by the Ethics Committee of Hospital Clinic de Barcelona (HCB/2020/1132), who waived the need for participant consent.

## Results

### Descriptive statistics for unmodeled hospitalizations and deaths

**Fig. 1:**
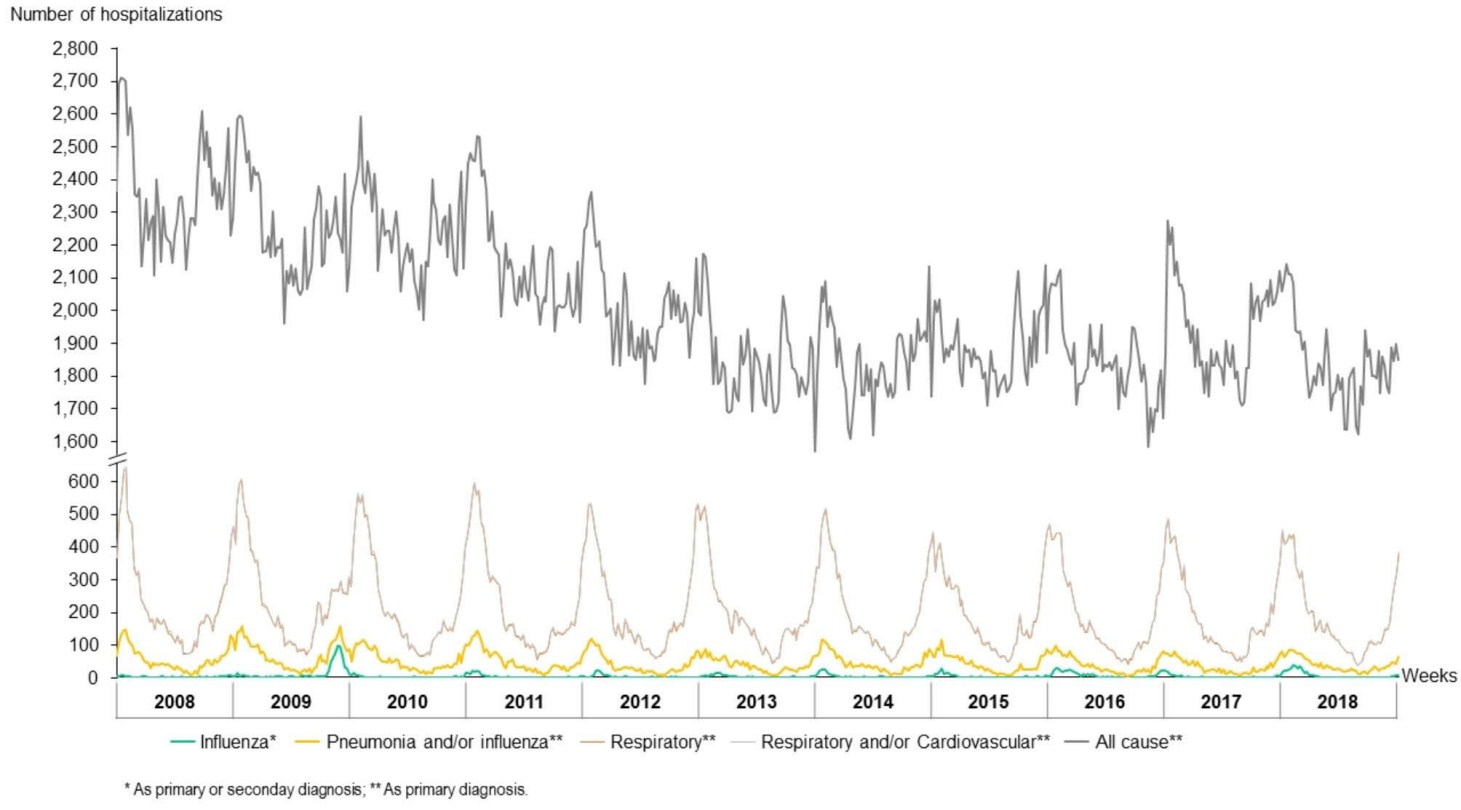
Weekly hospitalizations in children aged < 5 years old, by groups of ICD-9/10 codes, in Portuguese public hospitals, between 1st January 2008–31st December 2018

In 2018, Portugal had a resident population of 10.3 million, of which 4.2% (0.4 million) were aged < 5 years old [21]. During the study period, a total of 1.0 million all-cause hospitalizations were registered in children aged < 5 years old in Portuguese public hospitals. R&C, R, and P&I diagnoses accounted for 10.3%, 10.2%, and 2.2% of hospitalizations, respectively. The evolution of the weekly hospitalizations per groups of causes throughout the study period is displayed in Fig. 1. A total of 3,490 children aged < 5 years old have died in Portugal over the same period. R&C, R, and P&I diagnoses accounted for 3.7%, 2.2%, and 1.1% of deaths, respectively.

### Hospitalizations coded as due to Influenza

#### Hospitalization rate

A total of 2,319 influenza hospitalizations were registered in children aged < 5 years old between seasons 2008/09 and 2017/18, of which 843 (36.4%) during the last three seasons. The maximum number of influenza hospitalizations was observed in 2009/10, the H1N1 pandemic season. Excluding this atypical season, the mean annual number of hospitalizations per season was 189, corresponding to 41.3 cases per 100,000 children (Table 1). Influenza hospitalization rates decreased as age increased (Table 2). Influenza was the primary discharge diagnosis in 73.6%^1^ of the observed influenza hospitalizations, and a secondary discharge diagnosis in the remaining ones (Table 1).

**Table 1:** Number and characteristics of hospitalizations coded as due to influenza in children aged < 5 years old by epidemic season in Portuguese public hospitals between 2008/09 and 2017/18 seasons

Season	N	Rate^c^	Influenza as primary diagnosis (%)	Mean LoS (days)	In-hospital case fatality risk (%)	Use of supple-mental oxygen (%)	Use of non-invasive MV (%)	Use of invasive MV (%)	Mean hospitalization cost per patient^e^ (€)	With comor-bidities (%)
2008/ 2009	117	22.8	86.3	4.4	0.0	17.9	0.9	0.9	780	5.1
2009/ 2010	617	123.5	82.7	5.1	0.2	17.8	0.6	2.1	1,232	8.4
2010/ 2011	158	32.2	74.7	6.6	0.6	34.2	3.2	3.2	1,576	5.7
2011/ 2012	128	26.6	68.0	6.8	0.0	31.3	1.6	4.7	1,295	17.2
2012/ 2013	122	26.3	73.8	6.6	0.8	41.8	3.3	4.9	2,471	13.1
2013/ 2014	180	40.0	70.0	7.0	0.0	32.2	3.3	1.1	1,253	10.0
2014/ 2015	154	35.3	66.2	5.3	0.0	32.5	3.2	1.3	967	12.3
2015/ 2016	340	79.4	75.3	6.0	0.3	38.2	2.4	2.9	1,307	12.9
2016/ 2017	170	39.9	70.0	6.3	0.0	40.6	2.9	1.8	1,209	12.4
2017/ 2018	333	77.4	76.0	5.9	0.0	33.0	5.1	1.8	1,005	10.8
9-year mean^a^	189	41.3	73.6	6.1	0.2	34.3	3.1	2.4	1,278	11.2
3-year mean^b^	281	65.6	74.5	6.0	0.1	36.7	3.6	2.3	1,174	12.0

Abbreviations: LoS: Length of stay; MV: Mechanical ventilation; N: Number of hospitalizations coded as due to influenza

^a^ Includes the following seasons: 2008/09, 2010/11, 2011/12, 2012/13, 2013/14, 2014/15, 2015/16, 2016/17, 2017/18. Excludes season 2009/10, since it was affected by the 2009 influenza A (H1N1) pandemic; ^b^ Includes the most recent seasons, namely 2015/16, 2016/17, 2017/18; ^c^ Rate of hospitalizations coded as due to influenza per 100,000 children aged < 5 years old; ^d^ Discharges to long-term hospital care, specialized care, post-hospital care, palliative care, transference to other institution with inpatient care or home care; ^e^ Includes > 1 influenza hospitalization/patient

**Table 2:** Summary of the characteristics of the hospitalizations coded as due to influenza in children aged < 5 years old by age group and presence of comorbidities in Portuguese public hospitals between 2008/09 and 2017/18 seasons

Variable	Age Group (in months)									Aged < 5 years old with comorbidities	Aged < 5 years old without comorbidities
	0–5	6–11	12–23	24–35	36–47	48–59	< 24	24–59	0–59		
Share of hospitalizations (% of hospitalizations)											
9 years mean^a^	32.5	17.7	24.0	11.8	8.1	5.8	74.3	25.7	100.0	11.2	88.8
3 years mean^b^	30.4	18.7	25.1	12.2	7.5	6.0	74.3	25.7	100.0	12.0	88.0
**Hospitalization rate (hospitalizations per 100,000 children)** ^c^											
9 years mean^a^	139.4	75.7	50.3	24.5	16.5	11.6	78.6	17.4	41.3	NA	NA
3 years mean^b^	196.7	121.4	81.7	40.2	25.0	19.9	120.4	28.4	65.6	NA	NA
**Mean LoS (in days)**											
9 years mean^a^	6.1	6.3	6.4	5.7	6.4	4.6	6.2	5.7	6.1	12.8	5.2
3 years mean^b^	5.2	6.5	7.1	5.5	6.0	5.1	6.2	5.6	6.0	11.9	5.2
**In-hospital case fatality risk (% of hospitalizations)**											
9 years mean^a^	0.0	0.0	0.0	0.5	0.7	1.0	0.0	0.7	0.2	0.5	0.1
3 years mean^b^	0.0	0.0	0.0	0.0	0.0	2.0	0.0	0.5	0.1	0.0	0.1
**Use of supplemental oxygen (% of hospitalizations)**											
9 years mean^a^	28.3	44.2	39.4	35.3	30.4	19.2	35.7	30.1	34.3	48.7	32.4
3 years mean^b^	29.7	46.8	42.9	35.0	28.6	27.5	38.5	31.3	36.7	51.5	34.6
**Use of non-invasive mechanical ventilation (% of hospitalizations)**											
9 years mean^a^	5.2	2.7	1.2	2.5	3.6	1.0	3.3	2.5	3.1	7.9	2.5
3 years mean^b^	6.3	2.5	1.4	2.9	4.8	2.0	3.7	3.2	3.6	6.9	3.1
**Use of invasive mechanical ventilation (% of hospitalizations)**											
9 years mean^a^	3.6	2.0	1.7	1.5	1.4	3.0	2.6	1.8	2.4	9.9	1.5
3 years mean^b^	2.7	1.9	2.4	1.0	1.6	3.9	2.4	1.8	2.3	8.9	1.3
**Mean hospitalization cost per patient** ^d^ **(€)**											
9 years mean^a^	1,678	1,282	925	1,050	1,165	1,048	1,339	1,086	1,278	3,812	953
3 years mean^b^	1,150	1,513	1,008	1,171	940	1,095	1,194	1,086	1,174	2,612	972

Abbreviations: LoS: length of stay; NA: Not available

^a^ Includes the following seasons: 2008/09, 2010/11, 2011/12, 2012/13, 2013/14, 2014/15, 2015/16, 2016/17, 2017/18. Excludes season 2009/10, since it was affected by the 2009 influenza A (H1N1) pandemic; ^b^ Includes the most recent seasons, namely 2015/16, 2016/17, 2017/18; ^c^ Rate of hospitalizations coded as due to influenza per 100,000 children; ^d^ Includes > 1 influenza hospitalization/patient

### Demographic and clinical characteristics

The male to female ratio was 1.3, with boys accounting for 56.2% of influenza hospitalizations^1^. Children aged bellow 12 months of age accounted for 50.2% of hospitalizations (Table 2)^1^. Children without known predisposing risk factors for severe influenza infection accounted for 88.8% of influenza hospitalizations^1^ (Table 2). The share of influenza hospitalized children with a reported comorbidity increased with age. Comorbidities were observed in 7.8%, 10.3%, 9.5%, 15.4%, 17.4% and 23.2% of influenza hospitalizations in children aged 0–5, 6–11, 12–23, 24–35, 36–47 and 48–59 months of age, respectively^1^. Respiratory or lung disease was the most common comorbidity, closely followed by cardiovascular disease (**Figure ****S1**).

### Severity indicators

**Fig. 2:**
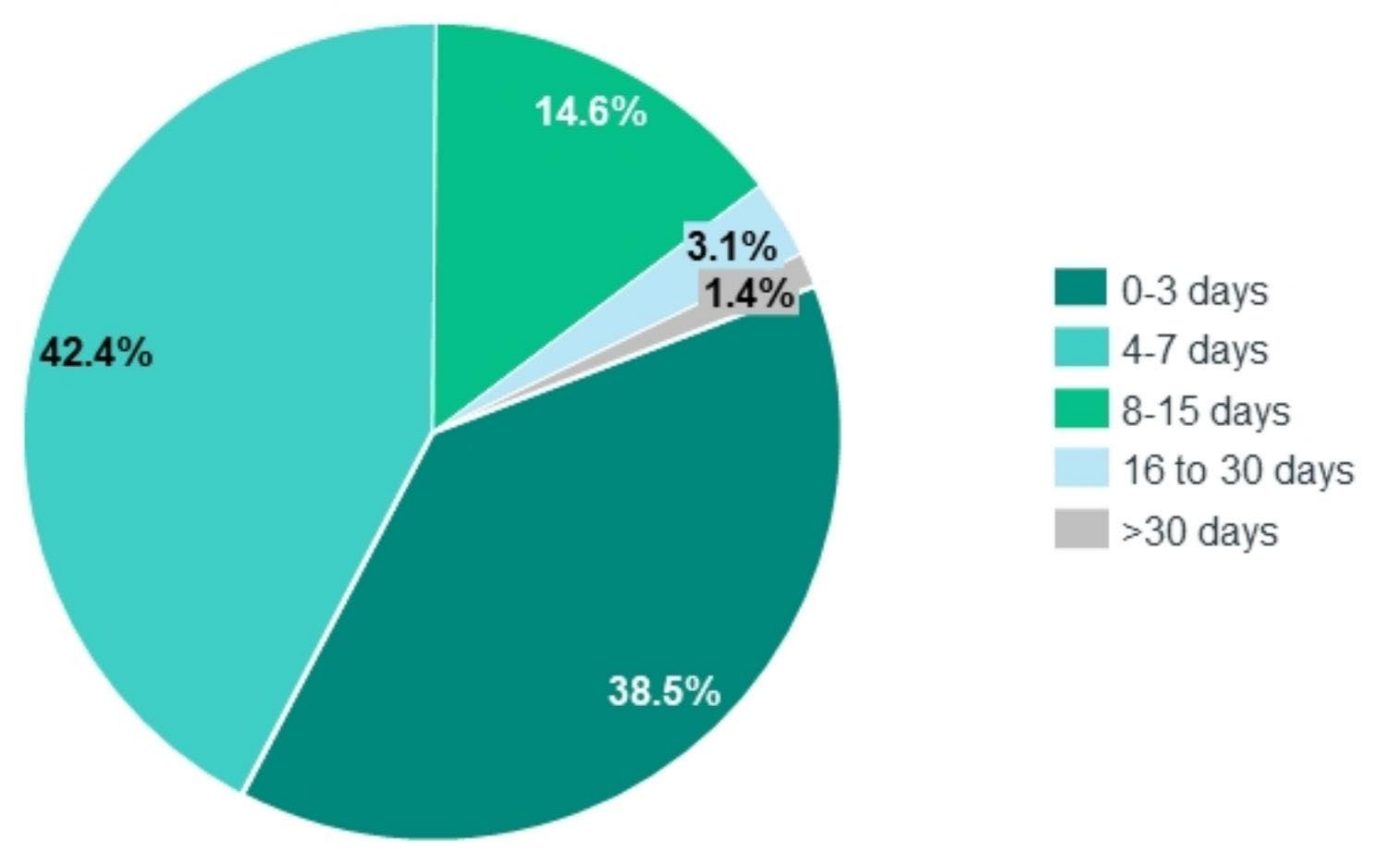
Percentage of hospitalizations diagnosed as due to influenza in children aged < 5 years old by length-of-stay at the hospital, in Portuguese public hospitals, between 2008/09 and 2017/18

The mean length of stay (LoS) stood at 6.1 days, increasing to 12.8 in children with comorbidities^1^ (Table 2). Most children (42.4%) were hospitalized during 4–7 inpatient days (Fig. 2) ^1^. The mean LoS was lower in children aged between 48 and 59 months of age (4.6 days) and ranged between 5.7 and 6.4 days in the other age groups^1^ (Table 2). Invasive mechanical ventilation was used in 2.4% of influenza hospitalizations, non-invasive ventilation in 3.1% and oxygen supplementation in 34.3%^1^ (Table 1). During the study period, four deaths were observed among the 2,319 influenza hospitalizations. Excluding the pandemic season, the mean in-hospital case fatality risk was 0.2%. On average, children with comorbidities had more severe influenza hospitalization episodes than those without comorbidities, measured by the LoS, use of mechanical ventilation, oxygen supplementation and in-hospital case fatality risk (Table 2).

### Direct cost

**Fig. 3:**
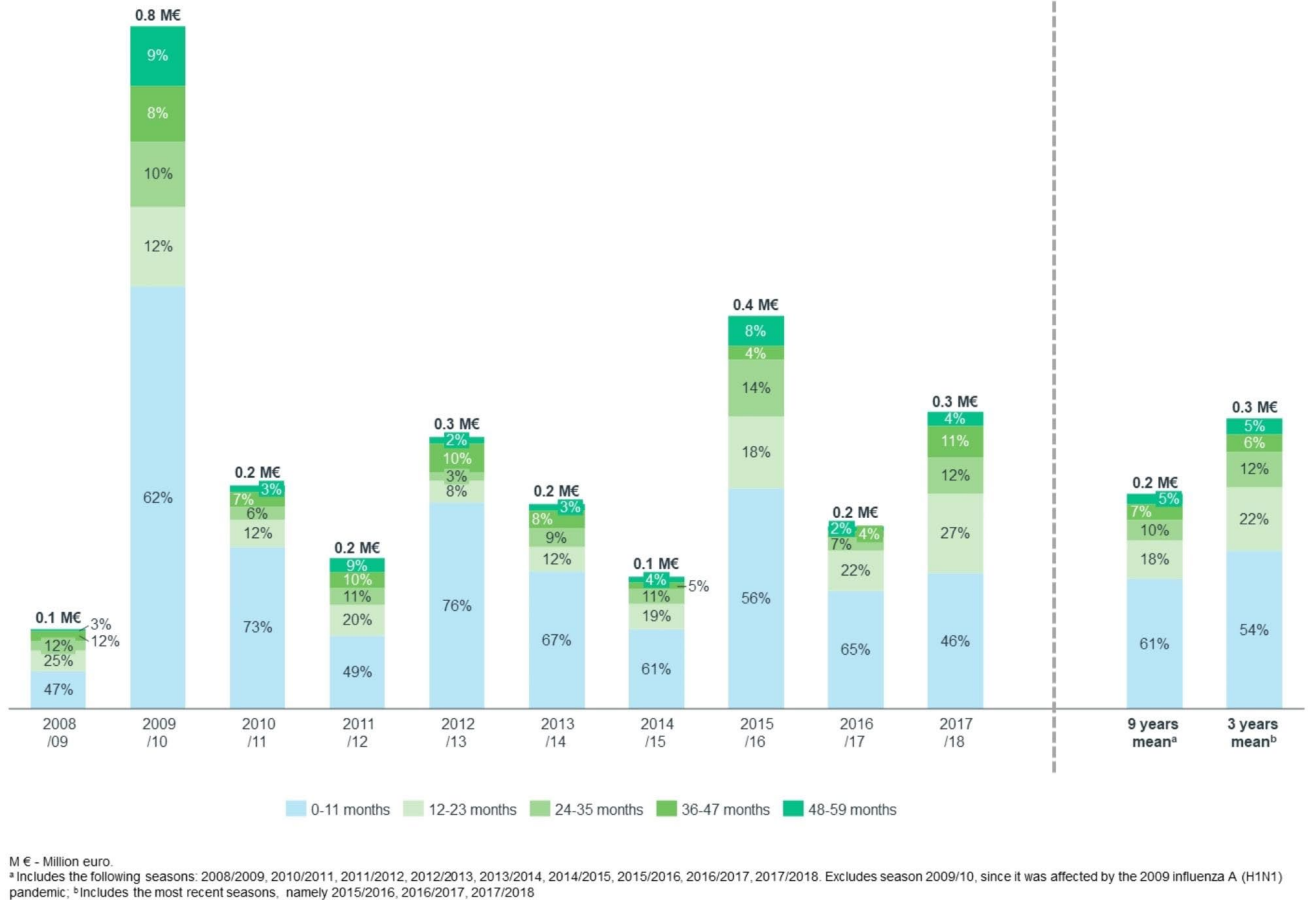
Direct cost of hospitalizations coded as due to influenza in children aged < 5 years old by months of age and epidemic season, in Portuguese public hospitals, between 2008/09 and 2017/18 (million €)

Over ten years, the 2,319 hospitalizations coded as due to influenza in children aged < 5 years old cost the Portuguese NHS €2.9 million. Costs varied considerably according to the epidemic season, from €0.1 million in 2008/09 to €0.8 million in 2009/10 (Fig. 3). The mean direct annual cost over nine seasons was €0.2 million^1^, of which 60.6%, 17.6%, 9.7%, 7.4% and 4.7% were generated by children aged 0–11, 12–23, 24–35, 36–47 and 48–59 months of age, respectively^1^ (Fig. 3). Children without registered comorbidities, accounted for 66.5% of all influenza direct hospitalization costs^1^. Mean per hospitalized children aged < 5 years old was €1,278 (Table 1)^1^. The mean cost of hospitalization was €953 in children without comorbidities, increasing fourfold in children with comorbidities (Table 1)^1^.

### Influenza-associated excess hospitalization

#### Estimated excess hospitalization

The influenza-associated excess hospitalizations in children aged < 5 years old between 2008 and 2018 were estimated at 1,850 in the P&I, 1,760 in the R, 1,787 in the R&C, and 1,879 in the all-cause model (Table 3). The all-cause hospitalizations model had an inferior performance, as detailed in Supplementary Materials (**Table S2**). The model using P&I hospitalizations estimated influenza-associated excess hospitalizations in all seasons, except for 2012/13. The absolute and relative influenza-associated excess hospitalizations is presented in Table 3 for all groups of diagnoses and seasons.

**Table 3:** Estimated influenza-associated excess hospitalizations in children aged < 5 years old, in absolute number of cases and per 100,000 children, by group of diagnoses and epidemic season in Portuguese public hospitals between 2008/09 and 2017/18 seasons

		2008/ 2009	2009/ 2010	2010/ 2011	2011/ 2012	2012/ 2013	2013/ 2014	2014/ 2015	2015/ 2016	2016/ 2017	2017/ 2018
P&I	N	119	570	183	40	-^b^	259	84	278	101	215
	95% CI	40–207	512–629	115–262	-28 to 103	-266 to -119	198–312	27–135	207–334	40–155	132–281
	Rate^a^	23.0	112.6	37.0	8.2	-^b^	56.8	19.0	64.4	23.8	50.1
R	N	-^b^	95	-^b^	-^b^	-^b^	367	-^b^	646	-^b^	652
	95% CI	-1313 to -658	-171 to 374	-1147 to -533	-412 to 99	-1740 to -1158	152–596	-1287 to -817	373–895	-382 to 109	351–929
	Rate^a^	-^b^	18.8	-^b^	-^b^	-^b^	80.5	-^b^	149.3	-^b^	152.2
R&C	N	-^b^	93	-^b^	-^b^	-^b^	381	-^b^	652	-^b^	662
	95% CI	-1339 to -689	-176 to 371	-1166 to -556	-405 to 105	-1754 to -1169	165–613	-1,292 to -820	377–909	-387 to 105	364–942
	Rate^a^	-^b^	18.3	-^b^	-^b^	-^b^	83.4	-^b^	150.8	-^b^	154.7
All-cause	N	-^b^	-^b^	922	214	-^b^	-^b^	-^b^	743	-^b^	-^b^
	95% CI	-2442 to -1097	-994 to 145	323–1576	-428 to 862	-4506 to -3112	-1718 to -516	-1934 to -928	72-1382	-1380 to -335	-1147 to 297
	Rate^a^	-^b^	-^b^	186.4	44.0	-^b^	-^b^	-^b^	171.8	-^b^	-^b^

Abbreviations: CI - Confidence interval (of the N); N - Number of excess hospitalizations; R – Respiratory; R&C - Respiratory or cardiovascular; P&I - Pneumonia or influenza

^a^ Rate of influenza-associated excess hospitalizations per 100,000 children aged < 5 years old; ^b^ No influenza-associated excess hospitalizations estimated in this season

#### Estimated direct cost from the excess hospitalization

The direct cost of influenza-associated excess hospitalizations in children aged < 5 years old between 2008 and 2018 was estimated at €1.8 million in the P&I, €1.5 million in the R, €1.6 million in the R&C, and €1.6 million in the all-cause model.

### Influenza-associated excess mortality

We estimated a total of 95 influenza-associated excess deaths in children aged < 5 years old between 2008 and 2018 in the all-cause model, 14 in the R&C, and 9 in the R models (Table 4). The analysis was not performed for the P&I diagnoses as the number of total P&I deaths (40 deaths between 2008 and 2018) was not sufficient to perform the statistical analysis. The performance of the models for each group of diagnoses is presented in Table S2. In this age group, the accuracy is higher in the all-cause deaths model.

**Table 4:** Estimated influenza-associated excess deaths in children aged < 5 years old, in absolute number of cases and per 100,000 children, by group of diagnoses and epidemic season in Portugal between 2008/09 and 2017/18 seasons

		2008/ 2009	2009/ 2010	2010/ 2011	2011/ 2012	2012/ 2013	2013/ 2014	2014/ 2015	2015/ 2016	2016/ 2017	2017/ 2018
P&I	N	-^c^									
	95% CI										
	Rate^a^										
R	N	4.0	0.5	4.7	-^b^	-^b^	0.0	-^b^	-^b^	-^b^	0.1
	95% CI	3.2–5.7	-1.0 to 2.1	4.1–7.1	-2.9 to 1.4	-2.9 to 2.0	-0.5 to 2.1	-0.9 to 1.6	-0.8 to 2.7	-1.8 to 1.2	1.3-5.0
	Rate^a^	0.8	0.1	0.9	-^b^	-^b^	0.0	-^b^	-^b^	-^b^	0.0
R&C	N	4.3	0.3	4.6	-^b^	-^b^	1.5	1.5	0.4	-^b^	1.1
	95% CI	2.6–5.9	-1.7 to 2.4	2.9–6.7	-3.2 to 2.6	-2.7 to 2.4	-0.2 to 3.6	0.4–3.4	-1.0 to 3.5	-1.4 to 2.1	0.3–4.8
	Rate^a^	0.8	0.1	0.9	-^b^	-^b^	0.3	0.3	0.1	-^b^	0.3
All-cause	N	9.2	7.8	7.0	21.0	8.4	9.0	7.4	15.5	6.4	3.1
	95% CI	-2.9 to 20.4	-3.5 to 19.8	-4.4 to 18.3	10.1 to 31.7	-4.0 to 20.9	-1.3 to 18.7	-1.4 to 15.8	4.1 to 25.7	-2.3 to 15.0	-9.5 to 15.6
	Rate^a^	1.8	1.5	1.4	4.3	1.8	2.0	1.7	3.6	1.5	0.7

Abbreviations: CI - Confidence interval (of the N); N - Number of excess deaths; R – Respiratory; R&C - Respiratory or cardiovascular; P&I - Pneumonia or influenza

^a^ Rate of influenza-associated excess deaths per 100,000 children aged < 5 years old; ^b^ No influenza-associated excess deaths estimated in this season; ^c^ Not estimated due to the low number of deaths in children aged < 5 years old in this group of diagnoses

The model has estimated influenza-associated excess all-cause deaths in all seasons. Season 2011/12 was estimated to have been the most fatal, with 21 (95% CI: 10–32) influenza-associated all-cause deaths in children aged < 5 years old (4.3 per 100,000 children). The absolute and relative influenza-associated excess deaths is presented in Table 4 for all groups of diagnoses and seasons.

## Discussion

This was the first study exploring the characteristics of children under five years of age that were hospitalized for influenza in Portugal, and estimating the morbidity and mortality caused by influenza over ten seasons. We found that influenza viruses led to a high number of hospitalizations in children, especially in those under 24 months of age and who were previously healthy. These results should support an evidence-based reflection on the adequate preventive measures to protect this age group.

### Influenza hospitalization rates

Excluding the 2009/10 H1N1 pandemic, the mean annual number of hospitalizations per season was 189, corresponding to 41.3 cases per 100,000 children, which is bellow general estimates for high-income countries [22]. Influenza hospitalization rates for Portugal were approximately half the estimates published for France [9] and Poland [23] – where differences may be partially explained by the included seasons and by cases treated in the emergency services, which were not accounted for in our study [9, 23] – and were also lower than the ones reported for Spain using the same methodology and period of analysis [11]. Future studies should explore the possible causes for these lower rates.

### Characteristics of children hospitalized due to Influenza

Three-fourths of hospitalized children were aged < 24 months. Unsurprisingly, hospitalization rates decreased with increasing age [24, 25]. Across all age groups, we found that most children hospitalized for influenza were previously healthy, which is consistent with studies from other European countries [9, 25]. The presence of comorbidities increased with age, ranging from 7.8% in children aged 0–5 months to 23.2% in children aged 48–59 months of age.

### Severity of hospitalizations

The study reveals a non-negligible use of mechanical ventilation during hospitalizations coded as due to influenza (2.4% invasive and 3.1% non-invasive ventilation), potentially reflecting the cases requiring care at the ICU. These are not far from those reported by Tillard et al. (2022) for France, where 2.2% of children aged < 24 months hospitalized for influenza were admitted to ICU [9]. Mean in-hospital case fatality risk was also within reported ranges [11, 23]. [14]

### Influenza-associated excess hospitalization and mortality

The study highlights the complexity of estimating the burden of influenza in children. [28]The total number of hospitalizations coded as due to influenza (2,319) over ten seasons was higher than the estimated influenza-associated excess hospitalizations in all groups of diagnoses – which ranged from 1,760 to 1,879 -, as the models were not able to detect influenza-associated excess hospitalizations in all seasons. Similar conclusions would be reached if we contrasted other published estimates with the hospitalizations coded as due to influenza obtained from our study, for the comparable years [26]. As expected, the excess morbidity and mortality estimates varied across the ten seasons and four groups of diagnoses. More studies are needed to establish which cause-specific data are more adequate when estimating morbidity and mortality due to influenza in children [27] and weekly ILI incidence rates specific for children aged < 5 years old are needed to improve the models’ performance, as different weekly trends in the virus circulations are expected across age groups [28].

Findings from our study raise the question on the extent of the under-detection of influenza amongst the younger Portuguese population. In 2015/16 and 2017/18 we estimated around 650 annual influenza-attributed excess R&C hospitalizations, almost the double from the ones coded as due to influenza, results which appear more aligned with international estimates on the rate of influenza hospitalizations [9, 22, 23]. Furthermore, all models estimated a higher number of influenza-associated excess deaths – ranging from 9 to 95 over the total period – in comparison with the four deaths that had been observed during the hospitalizations coded as due to influenza.

 [29]In terms of mortality, we have estimated a mean of 2.1 influenza-associated all-cause deaths per children aged < 5 years old^1^, which is lower than the one estimated by Nunes et al. (2.6) between 1980 and 2004 [29]. It is however higher than the one reported more recently by Nielsen et al. in the pooled estimates of mortality attributable to influenza in children aged < 5 years old in 24 European countries, which varied between 0.4 and 1.1 [30], and higher than the 1.5 reported by Pumarola et al. (2023) for Spain, using the same methodology and period of analysis [11]. Comparison of results with those from other studies is challenging as there are few studies reporting results of excess hospitalization and mortality modelling in children – in Portugal and in other European countries – and as they are sensitive to methodological and temporal differences.

Future studies would benefit from better surveillance data in this age group, with age-stratified data and with information on whether hospitalized children had been vaccinated. Generating more comprehensive evidence contributes to a better understanding of the implications that influenza may have to public health and may help to generate awareness and strengthen preventive policies.

### Economic burden of Influenza

 [4, 33–37]Although the mean cost of hospitalization was fourfold in children with comorbidities than in those without them, the total cost to the NHS of influenza hospitalizations was mainly driven by children without comorbidities. These results should be considered when assessing whether the current immunization strategies – targeting only high-risk children in Portugal - are sufficient to address the clinical and economic burden of influenza infection across all children and other indirectly affected age groups. Studies demonstrate that the vaccine effectiveness against influenza-related hospitalizations is high in children [31]. Moreover, children – especially schoolchildren, can also be a vehicle of transmission of the influenza virus across the community [3, 32]. Understanding the impact and cost-effectiveness from the extension of influenza vaccination to all children aged 6 months to 5 years of age put in place in the USA, Canada and by several European countries would be advisable [5, 7, 9, 33].

### Limitations

The results reported in this study should be interpreted considering its limitations. The administrative data from hospitalizations did not include laboratory data nor information on whether the children had been vaccinated for influenza. It is also subject to coding errors or missing information. Hospitalization data includes all NHS hospitalizations, but not those in the private sector. However, the mortality data are representative of the country, as all deaths registered in the nation were used.

The excess morbidity and mortality modeled with ILI cannot strictly be attributed to influenza alone as our time-series models used ILI incidence as an indicator of influenza activity, without additional control variables [18, 34]. [41]Other variables could have also been used, such as temperature and laboratory data on the circulation of other respiratory viruses [35, 36]. Future studies would benefit from considering other respiratory viruses, namely the respiratory syncytial virus which was estimated by Rodrigues et al. (2019) to have an impact on children all-cause mortality in Portugal twice as high as the one from influenza in season 2016/2017 [37]. Furthermore, as previously addressed, the age-stratified weekly ILI incidence rates were not available for the complete study period limiting the predictive capability of the models [28]. Another limitation from our study is that the age groups used in our time series models were not stratified for 0–23 and 24–59 months of age, although the first group represents most of the hospitalizations coded as influenza [38].

Regarding the economic burden of influenza, our study covers only the tip of the iceberg, as only direct costs of hospitalizations in the NHS were considered, thus excluding children treated in the private and in the outpatient setting, both increasing in recent years. Indirect costs of lost productivity from parents or caregivers were not computed, although they may be generated even by milder cases that do not require hospitalization [39].

## Conclusions

Results from the BARI study confirm the high burden of severe influenza in children under 5 years old in Portugal, mostly driven by those without comorbidities. The findings of this study suggest that strategies targeting only high-risk children will provide an inadequate protection and ability to reduce the clinical and economic burden of influenza infection.

### Electronic supplementary material

Below is the link to the electronic supplementary material.

Supplementary Material 1

## Data Availability

The data that support the study’s findings are available from IQVIA, but there are restrictions on their availability because they were used under license for the current study and thus are not publicly available. Data are, however, available from the authors upon reasonable request and with permission from IQVIA.

## Footnotes

1 Mean for nine seasons (2008/2009 to 2017/2018), i.e., excluding the H1N1pdm09 pandemic (2009/10).

## Declarations

### Authors’ information (optional)

NA.

## Abbreviations

BARI: Burden of Acute Respiratory Infections
CI: Confidence Interval
ICD: International Classification of Diseases
ILI: Influenza-like illness
INE: *Instituto Nacional de Estatística*
LoS: Length of stay
MV: Mechanical ventilation
NHS: National Health Service
P&I: Pneumonia and/or influenza
R: Respiratory
R&C: Respiratory and/or cardiovascular
