# Supplementary material for: Uncovering the burden of Influenza in children in Portugal, 2008–2018
Source: BMC Infect Dis. 2024 Jan 18;24:100. doi: 10.1186/s12879-023-08685-z (PMC10797867; doi:10.1186/s12879-023-08685-z)

### Table S1. Diagnostic codes used to identify comorbidities/risk factors for influenza

| Broader | Narrow | | | ICD-9 Diagnosis codes | | ICD-10 Diagnosis Codes |
| --- | --- | --- | --- | --- | --- | --- |
| Cardiopulmonary | | Chronic cardiac disease | 393.xx – 398, 410.xx – 417.xx; 420.xx – 429.xx | | I05.xx – I09.xx, I20.xx – I25.xx; I26.xx – 128.xx; I30.xx – I.52.xx; T80.0; T81.71; T81.72; T82.81 | |
|  |  | CVD | 430 - 438.xx | | I60-I69; G45-G46; | |
|  |  | Hypertension | 401.xx – 405.xx | | I11.0, I11.9, I16.0, I16.1, I16.9 | |
|  |  | Peripheral vascular disease | 443.9 | | I73.9 | |
|  |  | High risk congenital heart disease | 745.0 - 745.4; 745.6x - 745.8; 746.01 - 746.5; 746.7 - 746.85; 746.87; 747.1x; 747.21 - 747.49 | | Q20-Q26 | |
|  |  | Low risk congenital heart disease | 745.5; 745.9; 746.00; 746.6; 746.86; 746.89; 746.9; 747.0; 747.20; 747.83 | | P29.3; Q20.9; Q21.1; Q21.9; Q22.3; Q23.3;  Q23.8; Q23.9; Q24.6; Q24.8; Q24.9; Q25.0; Q25.4 | |
|  |  | BDP | 770.7x | | P27 | |
| Respiratory/Lung disease | | Bronchiectasis | 494, 748.61, 494.0, 011.5 | | J47.0, J47.1, J47.9, Q33.4 | |
|  |  | Interstitial pulmonary fibrosis of prematurity, Wilson-Mikity syndrome | 770.7x | | P 27 | |
|  |  | Wilson-Mikity syndrome | 770.7x | | P27.0 | |
|  |  | Congenital anomalies of respiratory system | 748., | | Q30-Q34 | |
|  |  | Chronic perinatal respiratory disease | 770.7x | | P28 | |
|  |  | Other lung pathologies | 507.1; 514; 516.8; 517.2 - 517.8 | | J69, J84.09, M34.8 | |
|  |  | Cystic fibrosis | 277.0x | | E84 | |
|  |  | Asthma | 493.xx | | J45.xx (J45.20, J45.21, J45.22, J45.30, J45.31, J45.32, J45.40, J45.41, J45.42, J45.50, J45.51, J45.52, J45.901, J45.902, J45.909, J45.991, J45.998) | |
|  |  | Chronic obstructive pulmonary disease (COPD) | 490-492.x; 496 | | J40, J41.0, J41.1, J41.8, J42, J43.0, J43.1, J43.2, J43.8, J43.9, J44.0, J44.1, J44.9 | |
|  |  | Post inflammatory pulmonary fibrosis, Chronic and other pulmonary manifestations due to radiation, Chronic respiratory disease arising in the perinatal period, Idiopathic fibrosing alveolitis, With pulmonary manifestations | 515, 508.1, 770.7, 516.3, 277.02 | | J84.10, J70.1, P27, J84.112, E84.0 | |
|  |  | Rheumatoid lung | 714.81 | | M05.1 | |
| Immunocompromised | | HSCT | 996.88, 996.85, 41.04 - 41.09 | | T86.5. | |
|  |  | Lung transplant | 996.84; V42.6, 32.3x - 32.5x; 33.5x | | Z94.2, T86.83  T86.81, | |
|  |  | HIV | 042.xx | | B20 | |
|  |  | Hematologic malignancy (leukemia, lymphoma, multiple myeloma) | 203.xx - 208.xx; 238.4; 238.72 - 238.76; 289.83 | | C81-C96 | |
|  |  | Non-lung solid organ transplant | 199.2; 996.52; 996.55; 996.80 - 996.83; 996.86 - 996.89; E878.0; V42.0 - V42.3; V42.7; V42.83; V42.84; V45.87; V58.44, 07.94; 37.51; 41.94; 46.97; 50.51; 50.59; 52.80; 52.82; 52.83; 55.53; 55.69 | | Z94.0, Z94.1, Z94.4, Z94.5, Z94.7, T86.1, T86.2, T86.4, Z98.85, Z48.288, Z48.298, | |
|  |  | Hereditary haemolytic anaemias | 282.xx | | D55.xx – D59.xx | |
|  |  | Other immune deficiencies | 279.xx | | D80.xx – D89.xx | |
| Other comorbidities | | Down syndrome with CHD | 758.0 (down syndrome) | | Q90 | |
|  |  | Neuromuscular impairment | 330.x; 335.xx; 343.x; 356.x; 358.1; 359.0 - 359.23 | | E75.0; E75.1; E75.2; E75.4; F84.2; G12; G31.8; G31.9; G60; G71.0-G71.3; G80 (excl. G80.3); G93.8; G93.9 | |
|  |  | Diabetes Mellitus | 250.xx | | E08- E13 | |
|  |  | Chronic liver disease | 571.xx | | K70.xx – K77.xx | |
|  |  | Chronic kidney disease | 403.00, 403.1, 585.1, 585.2, 585.3, 585.4, 585.5, 585.6, 585.9 | | I12.0, I12.9, N18.1, N18.2, N18.3, N18.4, N18.5, N18.6, N18. | |

### Table S2. Performance of the excess hospitalization and excess mortality models in children aged <5 years old by group of diagnoses

| Model | Pneumonia and/or influenza | | Respiratory | | Cardiovascular and/or respiratory | | All-cause | |
| --- | --- | --- | --- | --- | --- | --- | --- | --- |
|  | r | MAPE | r | MAPE | r | MAPE | r | MAPE |
| Excess hospitalizations | 96% | 16% | 97% | 10% | 97% | 10% | 85% | 3% |
| Excess deaths | -^a^ | - | 64% | 53% | 50% | 45% | 41% | 21% |

r – Person’s correlation; MAPE; Mean absolute percentage error.

1. Not estimated due to the low number of deaths classified as due to pneumonia and or influenza in this age group.

### Figure S1. Percentage of hospitalizations diagnosed as due to influenza in children aged <5 years old in which the hospitalized children had a risk factor, by risk factor, in Portuguese public hospitals, between 2008/2009 and 2017/2018


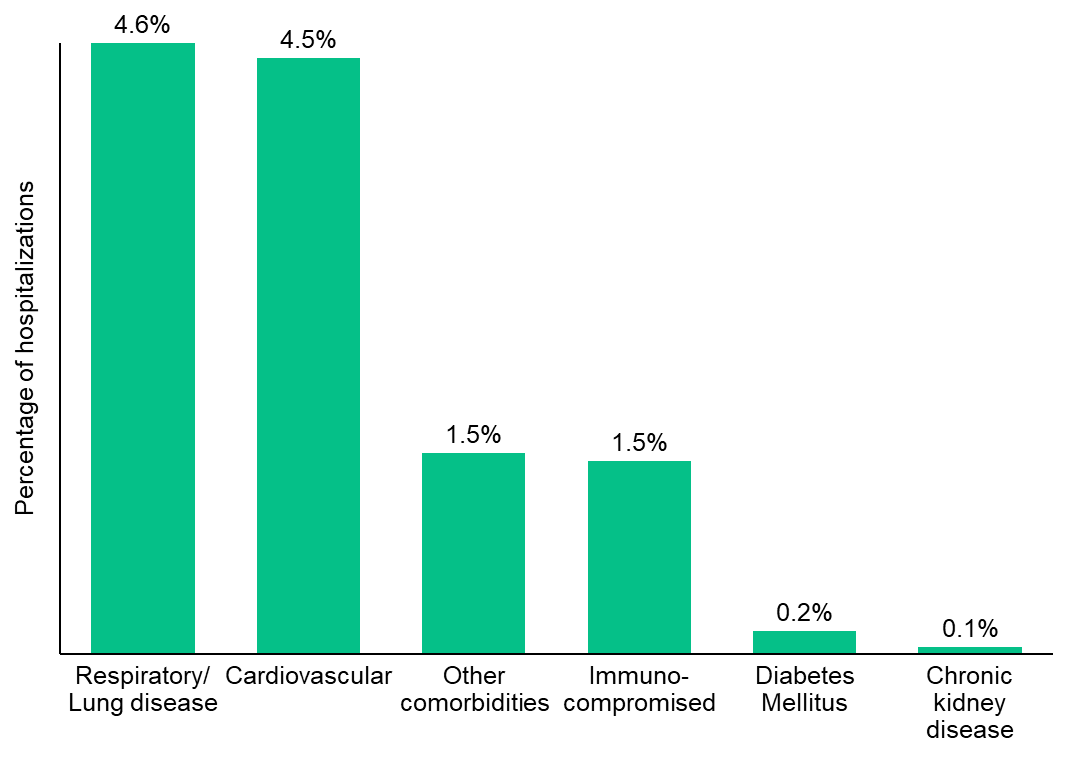

Supplement: Supplementary file 1 — Supplementary Material 1 [file 12879_2023_8685_MOESM1_ESM.docx]
